# A Case of Amitriptyline-induced Myocarditis

**DOI:** 10.7759/cureus.2840

**Published:** 2018-06-19

**Authors:** Thamer Kassim, Toufik Mahfood Haddad, Amandeep Rakhra, Amjad Kabach, Ahmad Qurie, Mohammad Selim, Ali S Nayfeh, Ahmed Aly, Mark J Holmberg

**Affiliations:** 1 Internal Medicine, Creighton University Medical Center, Omaha, USA; 2 Cardiology, Creighton University, Omaha, USA; 3 Internal Medicine, Creighton University, Omaha, USA; 4 Cardiovascular, CHI Creighton University, Omaha, USA; 5 Internal Medicine, Creighton University School of Medicine, Omaha, USA; 6 Radiology, Creighton University, Omaha, USA; 7 Cardiology, Creighton University, Omha, USA

**Keywords:** amitriptyline overdose, myocarditis, amitriptyline myocarditis, amitriptiline toxicity, drug induced myocarditis

## Abstract

Amitriptyline is a widely prescribed tricyclic antidepressant (TCA) with a very concerning cardiotoxicity profile, but it is one that has not been discussed much in literature. Here, we present a case of amitriptyline toxicity presenting as myocarditis with pericardial involvement. A 21-year-old male with no previous cardiac history presented to the emergency department (ED) with a decreased level of consciousness after an amitriptyline overdose as a suicidal attempt. For concerns with airway protection, the patient was intubated and subsequently admitted to the intensive care unit (ICU). An electrocardiogram (EKG) showed sinus tachycardia, prolonged QRS complex, prolonged QTc interval, and nonspecific ST-T wave changes. Intravenous fluid resuscitation and sodium bicarbonate were administered with a target blood pH of 7.5 to 7.55. Two days later, the patient was taken off mechanical ventilation and improved clinically. However, troponin levels began to rise with a peak level of 4.08 µg/L. He then began having fevers, elevated white blood cell counts (WBCs), and elevated inflammatory markers. Transthoracic echo (TTE) revealed an ejection fraction (EF) of 45%-50%, no wall segment motion abnormalities, and a mild-to-moderate pericardial effusion. Cardiac magnetic resonance (CMR) was done, which revealed changes indicative of acute myocarditis, moderate pericardial effusion, a calculated EF of 45% with a moderate left ventricular dilation, and no coronary artery stenosis or anomalous coronary artery origin. Given the patient’s age, the absence of cardiac risk factors, and the presence of an amitriptyline overdose along with his EKG, TTE, and CMR findings, we hypothesize that this myocarditis with pericardial involvement is due to amitriptyline-induced direct toxicity.

## Introduction

Tricyclic antidepressants are known to cause cardiotoxicity, resulting in lethal arrhythmias and sudden cardiac death. Amitriptyline-induced toxic myocarditis and dilated cardiomyopathy have been reported only once in the literature after autopsy [1]. The underlying pathophysiology of amitriptyline-induced cardiomyopathy is still not clear. Given the wide use of amitriptyline as an antidepressant medication, it is important to discuss this case of its overdose presenting as myocarditis with pericardial involvement and provide a brief review of amitriptyline-induced cardiotoxicity.

## Case presentation

A 21-year-old male with a previous medical history of depression and no other medical comorbidities presented to the emergency department (ED) with a decreased level of consciousness after taking an amitriptyline overdose as a suicidal attempt. The patient was found to have a Glasgow Coma Scale (GCS) of three and was subsequently intubated and admitted to the intensive care unit (ICU).

Initial laboratory workup showed lactic acidosis, negative troponin, and normal kidney and liver functions. An arterial blood gas (ABG) was done, and the patient was found to have metabolic acidosis (pH 7.2) with respiratory compensation. The EKG showed a wide complex tachycardia with a ventricular rate of 146 bpm, a QRS complex duration of 118 msec, and a prolonged QTc at 576 with nonspecific ST-T wave changes. The initial transthoracic echo (TTE) revealed a preserved ejection fraction (EF) at 65% and no wall segment motion abnormalities. The patient was started on intravenous fluids and intravenous sodium bicarbonate with a target pH of 7.5-7.55. On day two of admission, our patient improved clinically and was taken off mechanical ventilation. The QRS complex and QTc began to shorten. However, cardiac troponin I levels started to rise with a peak of 4.08 µg/L. The patient developed a fever with a maximum body temperature of 312.1 K, an elevation in WBC count at 13.2 x 109/L (with an absence of peripheral eosinophilia), and an elevation in brain natriuretic peptide at 399 pg/ml. Erythrocyte sedimentation rate and C-reactive protein were also elevated at 46 mm/hr and 18 mg/L, respectively. Reviewing the history further, the patient reported the ingestion of 41 amitriptyline 50 mg tablets. He denied having any recent flu-like symptoms, no exposure to sick contacts, and a viral panel was negative for common viruses, including coxsackie and adenovirus. His only prescribed medication was amitriptyline and he did not use over-the-counter medications regularly. Amitriptyline levels were not obtained as the patient was admitted while fully conscious after ingesting 41 tablets; this was confirmed through a tablet count of his prescription bottle.

**Figure 1:**
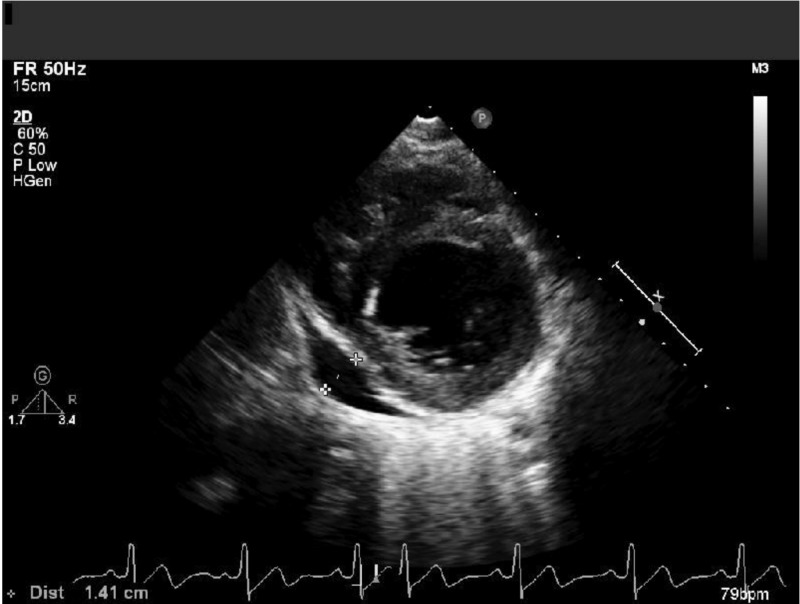
Short axis parasternal view showing a moderate-sized pericardial effusion

Cardiology service was consulted. Repeat TTE showed a mildly reduced EF at 45%-50%, mild to moderate pericardial effusion, and no wall segment motion abnormalities (Figure 1).

**Figure 2:**
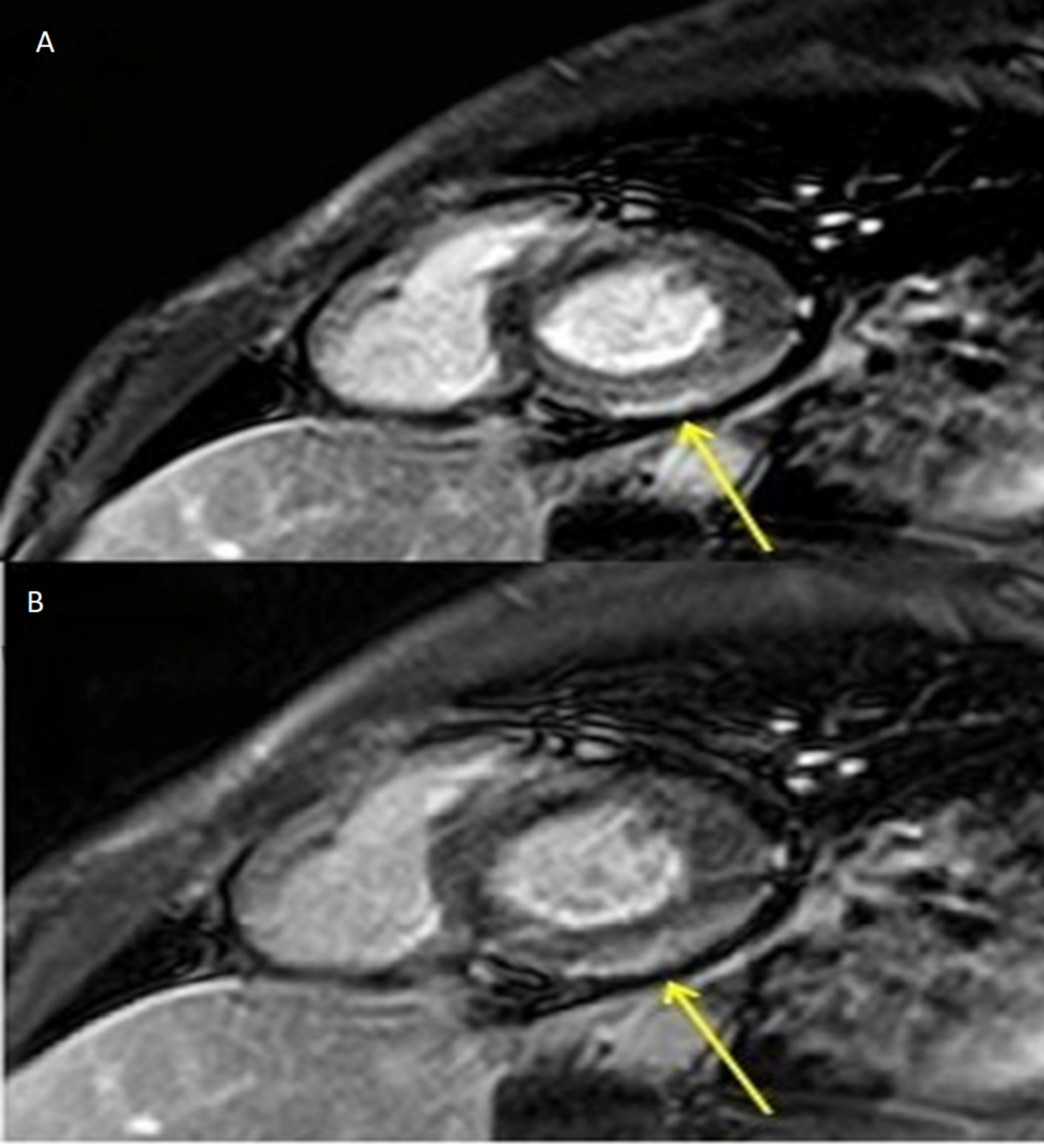
Short axis 10 m (A) and 15 m (B) delayed enhancement CMR images showing subtle enhancement (arrows) of the basal inferolateral epicardium CMR: cardiac magnetic resonance

**Figure 3:**
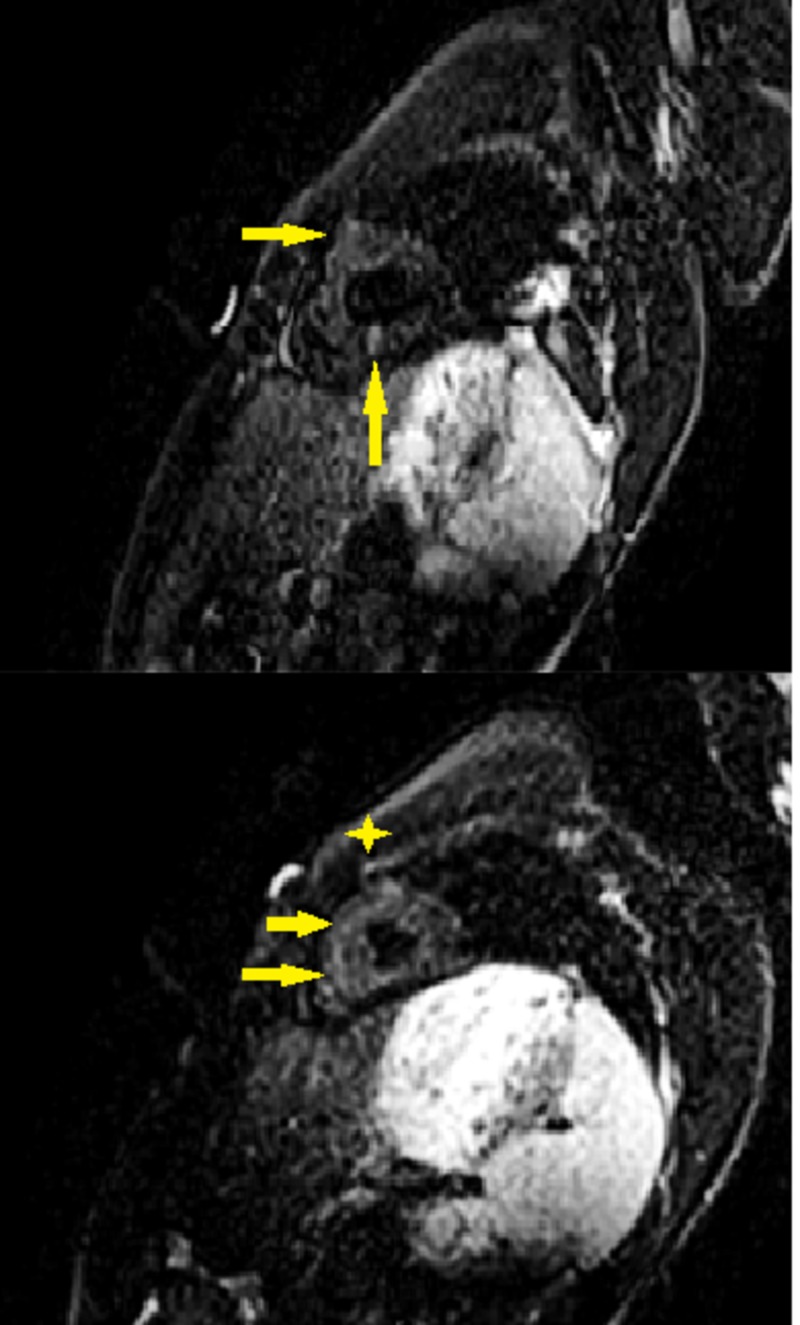
Short axis short TI inversion recovery (STIR) images showing scattered areas of increased signal intensity/edema (arrows) throughout the myocardium compared to skeletal muscles (asterisk). Note that this doesn’t follow territorial distribution.

Cardiac magnetic resonance (CMR) was done for a suspicion of acute myocarditis and revealed a moderately dilated left ventricle with mildly reduced EF at 45%, subtle enhancement of the basal inferolateral epicardium on delayed enhancement images (Figure 2), non-territorial scattered areas of edema within the myocardium (Figure 3), and moderate pericardial effusion. Findings were compatible with acute myocarditis. CMR was negative for coronary artery stenosis or an anomalous coronary artery origin as possible causes of ischemia or the elevated troponin level.

**Figure 4:**
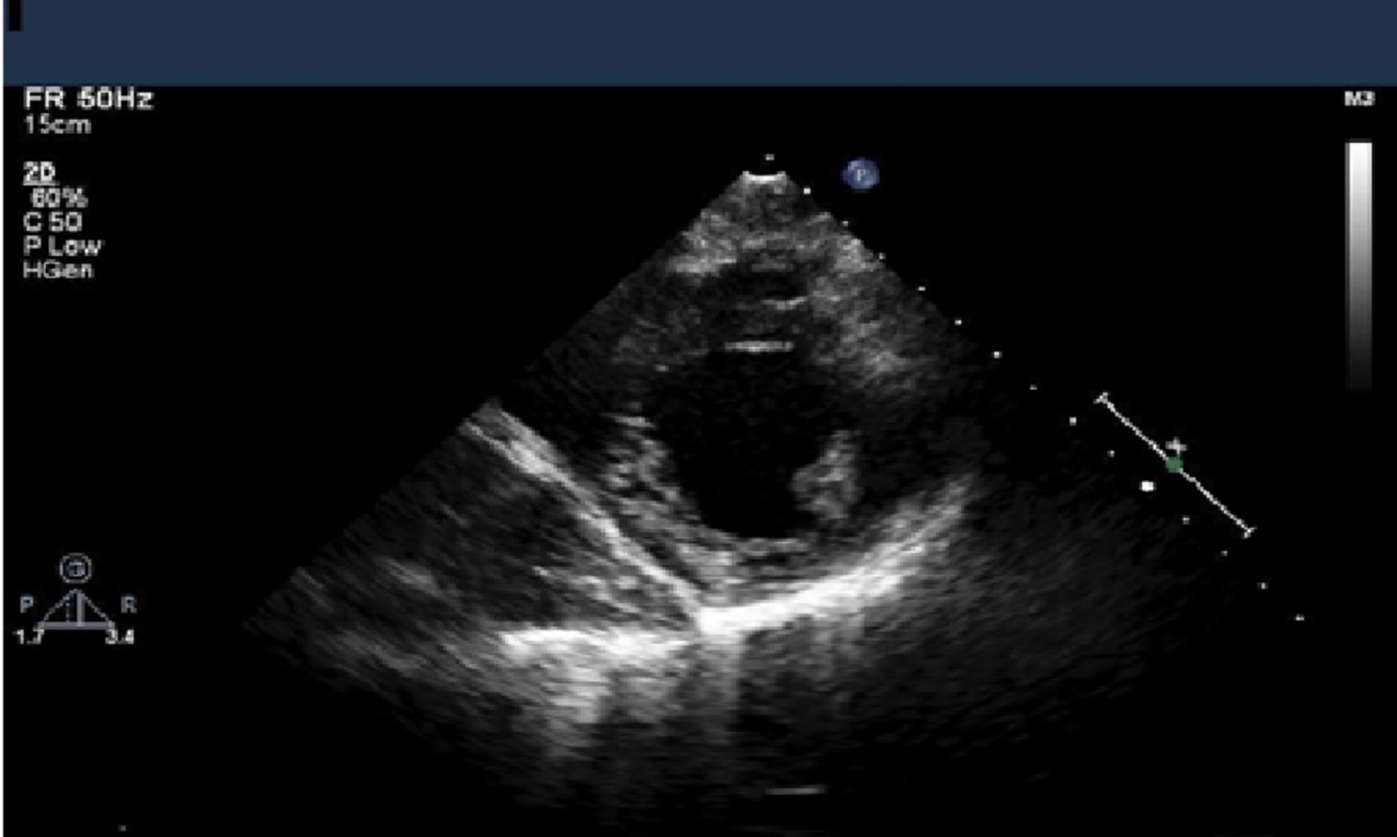
Short axis parasternal view showing a resolved pleural effusion

The patient was diagnosed with amitriptyline-induced cardiotoxicity in the form of drug-induced myocarditis with pericardial involvement. Supportive therapy with intravenous fluids, sodium bicarbonate, and the correction of electrolytes contributed to the clinical improvement. The patient recovered well and was discharged home after seven days of hospitalization. On the one month follow-up, the troponin level was repeated and was within normal limits. Repeat TTE demonstrated a normal left ventricular function with an EF of 65% and resolved pericardial effusion (Figure 4).

## Discussion

Myocarditis is an inflammatory disease of the myocardium that involves the infiltration of the heart muscle with inflammatory cells triggering an inflammatory cascade. This may result in myocardial necrosis and ultimately fibrosis [2]. Myocarditis is a relatively common, yet challenging, cardiac condition to diagnose due to its variable presentation that can overlap with a variety of cardiac diseases [2]. Clinical manifestations can range from minor symptoms of fatigue to more critical presentations of chest pain, cardiogenic shock, arrhythmias, and sudden death. The etiology of myocarditis can be an infection (viral being the most common), autoimmune-mediated (i.e. cardiac sarcoidosis, giant cell myocarditis), or drug-induced [2]. Such causes of myocarditis result in inflammation at a cellular level, making it extremely hard to distinguish the etiology of the disease without a tissue biopsy [3]. In this case of drug-induced myocarditis, it is proposed that amitriptyline may cause damage by either hypersensitivity or by direct damage to the myocytes – also known as toxic myocarditis. Infiltration can be focal or diffuse, involving the whole myocardium, leading to the dilation of the involved segments. The type of infiltration and its severity play an important role in the prognosis and development of complications [3].

The gold standard for the diagnosis of myocarditis is by endomyocardial biopsy (EMB) and histology. Dallas criteria specify certain histological findings that need to be met to fit the criteria for diagnosis [3-4]. Although myocarditis is considered a histological diagnosis, it is typically diagnosed clinically in patients with or without cardiac disease who exhibit a combination of the following: a rise in cardiac biomarkers; electrocardiographic changes suggestive of acute myocardial injury; arrhythmias; and TTE or CMR findings indicative of the disease [2].

The role of echocardiography in the setting of acute myocarditis is helpful in excluding other possible etiologies of heart disease. There are no specific findings for acute myocarditis on TTE but the presence of left ventricular dysfunction and pericardial effusion are common and favor the diagnosis [4]. In recent years, CMR has become more popular in diagnosing myocarditis in light of it being a noninvasive procedure. While most EMBs are obtained from the right ventricular side of the interventricular septum, myocarditis starts as a focal process involving the epicardium of the left ventricular free wall, resulting in EMB sampling errors. The focal process of myocarditis can be visualized early on CMR, making it a more favorable modality for diagnosis [5]. Findings on CMR suggestive of acute myocarditis are based on “Lake Louise criteria,” which include a regional or global increased T2 signal intensity in the myocardium compared to the skeletal muscles, a global increase in early myocardial gadolinium enhancement, and at least one focus of delayed gadolinium enhancement in the non-ischemic pattern. In the setting of clinically suspected myocarditis, having two out of those three criteria on 1.5 T MRI is sufficient for making the diagnosis [6].

Cardiovascular effects secondary to an amitriptyline overdose is a major concern, especially in patients who have significant cardiac risk factors and comorbidities. The drug is highly concentrated in the myocardium and can cause cardiotoxicity through different mechanisms [7]. Initially, cardiotoxicity may appear as sinus tachycardia on EKG. With time, a broad-complex tachyarrhythmia may develop with a widening of the QRS complex, a prolongation of the QT interval, and nonspecific ST wave changes [7-8]. This is due to its effect on atrioventricular conduction through the blockage of fast sodium channels and the inhibition of potassium channels [9]. Without intervention, fatal dysrhythmias, such as bradycardia with second or third-degree heart block, asystole, and even sudden cardiac death may occur [8-10].

Amitriptyline has been associated with impaired cardiac contractility, especially when taken in high doses. The hypersensitivity of myocytes may lead to myocardial inflammation, potentially involving the pericardium and presenting with signs and symptoms of myocarditis and pericardial effusion [11]. These events can, in turn, cause direct myocardial depression and impaired myocardial contractility with a dilation of the ventricles and reduced EF.

Amitriptyline interferes with the reuptake of norepinephrine and direct myocardial depression, playing a role in decreased myocyte contractility, causing prolongation of the QT interval, and predisposing to torsade de pointes. Furthermore, it has anticholinergic and alpha-adrenergic blockade properties, which can cause hypotension and further worsen systemic perfusion [9-12]. The early initiation of therapy is vital for the retrieval of cardiac function. Typically, sodium bicarbonate has been found to reverse toxicity effects, with an end goal of blood pH of 7.5-7.55 and no regard to arterial pH at presentation. The administration of sodium bicarbonate was associated with volume expansion and improvement in systolic blood pressure, preventing the further development of acidosis. Also, the induction of hypokalemia by bicarbonate causes membrane hyperpolarization. Such an effect can lower the threshold voltage at which sodium channels open, which, in turn, diminishes the effect of sodium channel blockade. These changes can help in narrowing the QRS complex. Additionally, the withdrawal of all cardiotoxic offending agents and QTc prolonging medications in patients with TCA toxicity is very important [13-15].

Our patient is a 21-year-old male with no previous medical comorbidities and at low risk for cardiac sequelae. He claimed to ingest 41 tablets of amitriptyline 50 mg. His weight at admission was 67 kilogram (kg), putting him at a toxicity dose of approximately 30.6 mg/kg. EKG changes correlated with TCA-induced cardiotoxicity. CMR showed no evidence of coronary artery occlusion, and it was thought that the troponin elevation was due to amitriptyline’s direct toxic damage on cardiac myocytes. In addition, the patient’s low-grade fever, elevated WBC count (with no peripheral eosinophilia), elevation in inflammatory markers along with TTE and CMR findings were all suggestive of acute myocarditis with pericardial involvement. After excluding other causes, including viral infections, the use of other cardiac-offending medications, and the absence of peripheral eosinophilia, we hypothesized that the direct toxicity of amitriptyline was the cause of myocarditis, primarily leading to a mildly dilated cardiomyopathy in this patient. Although EMB is the gold standard for the diagnosis of myocarditis and cardiomyopathy, a biopsy was not done due to the invasiveness of the procedure. The patient’s clinical picture and CMR findings were enough to suggest the diagnosis of amitriptyline -induced myocarditis. Starting sodium bicarbonate and supportive therapy early, at the time of presentation, along with the withdrawal of cardiac-offending agents are the mainstay of therapy. Outpatient follow-up is important to assess the reversibility of the damage caused by the offending agent and to monitor improvements in EF.

## Conclusions

Amitriptyline toxicity is life-threatening and can cause acute myocarditis in addition to the known cardiotoxic profile of tricyclic anti-depressant medications. Physicians should be aware of this rare entity as a differential diagnosis for myocarditis with an unknown etiology.
